# Supplementary material for: Increased Pre-Operative Lung Immune Prognostic Index Score Is a Prognostic Factor in Cases of Pathological T3 Renal Cell Carcinoma
Source: Curr Oncol. 2025 Jun 7;32(6):335. doi: 10.3390/curroncol32060335 (PMC12192035; doi:10.3390/curroncol32060335)
Supplement: Supplementary file 1 [file curroncol-32-00335-s001.zip › Table S1.pdf]

Supplementary Table S1

The results of dNLR, LDH and LIPI (pre- and post-operatively) in 80 patients and the change in LIPI scores.

Patient No.	Pre-op dNLR	Pre-op LDH	Pre-op LIPI	Post-op dNLR	Post-op LDH	Post-op LIPI	Score change
1	1.75	191	0	1.14	158	0	
2	1.94	176	0	0.73	184	0	
3	3.45	188	1	3.07	119	1	
4	2.42	167	0	3.71	292	2	up
5	1.56	165	0	1.07	202	0	
6	1.81	177	0	1.56	168	0	
7	1.25	162	0	1.03	167	0	
8	3.02	156	1	2.44	155	0	
9	4.75	172	1	1.06	186	0	
10	2.34	206	0	1.86	203	0	
11	1.64	193	0	4.66	218	1	up
12	1.40	159	0	1.86	125	0	
13	2.15	174	0	2.4	194	0	
14	1.16	156	0	1.41	168	0	
15	1.26	224	1	1.29	192	0	
16	1.91	145	0	1.76	172	0	
17	2	202	0	1.09	160	0	
18	1.59	151	0	1.33	157	0	
19	1.91	170	0	1.59	168	0	
20	1.82	197	0	2.70	255	1	up
21	1.35	196	0	1.24	213	0	
22	1.92	156	0	1.06	179	0	
23	2.38	121	0	2.5	137	0	
24	2.52	178	0	1.64	207	0	
25	2	177	0	1.66	147	0	
26	1.85	239	1	1.85	211	0	
27	1.56	177	0	1.68	204	0	
28	1.42	180	0	1.09	168	0	
29	1.55	184	0	1.45	154	0	
30	1.52	234	1	1.18	198	0	
31	1.21	183	0	1.68	158	0	
32	2.31	160	0	2.25	176	0	
33	2.07	263	1	1.38	141	0	
34	4.38	163	1	2	108	0	
35	1.32	223	1	1.02	238	1	
36	1.7	164	0	1.84	166	0	
37	2.25	212	0	0.88	182	0	
38	1.29	134	0	1.17	121	0	

39	2	164	0	1.25	149	0	
40	2.25	140	0	1.53	140	0	
41	2	217	0	1.09	250	1	up
42	3.04	156	1	4.87	399	2	up
43	2	281	1	2.35	167	0	
44	3.62	176	1	3.04	219	1	
45	2.5	82	0	1.5	69	0	
46	0.42	184	0	0.45	133	0	
47	4.11	253	2	3.46	176	1	
48	2.4	180	0	1.61	177	0	
49	2.17	169	0	1.57	161	0	
50	2.78	144	0	2.47	124	0	
51	3.30	147	1	1.19	153	0	
52	1.86	202	0	1.52	155	0	
53	1.85	146	0	1.21	140	0	
54	1.52	176	0	1.28	187	0	
55	3.37	226	2	2.05	155	0	
56	1.73	180	0	1.09	158	0	
57	1.20	186	0	1.7	200	0	
58	3.68	244	2	2.47	192	0	
59	1.52	179	0	2	164	0	
60	1.15	461	1	0.51	205	0	
61	1.84	130	0	2.21	139	0	
62	1	194	0	1.28	183	0	
63	1.89	238	1	2.1	170	0	
64	1.52	180	0	1.38	164	0	
65	1.23	180	0	1.52	167	0	
66	2.31	163	0	3.04	169	1	up
67	1.38	147	0	1.27	132	0	
68	2.30	167	0	2	176	0	
69	1.16	151	0	0.93	157	0	
70	1.57	174	0	1.75	165	0	
71	2.6	186	0	3.33	162	1	up
72	1.54	168	0	1.5	179	0	
73	1.10	165	0	1.06	173	0	
74	2.93	162	0	0.82	193	0	
75	2.75	153	0	2.77	311	1	up
76	1.12	171	0	0.66	181	0	
77	2.05	186	0	1.60	145	0	
78	1.54	225	1	2.44	221	0	
79	1	181	0	1	162	0	
80	3.53	335	2	1.85	228	1	

dNLR, derived neutrophil-to-lymphocyte ratio; LDH, lactate dehydrogenase; LIPI, Lung immune prognostic index.
