# Supplementary material for: Increased Pre-Operative Lung Immune Prognostic Index Score Is a Prognostic Factor in Cases of Pathological T3 Renal Cell Carcinoma
Source: Curr Oncol. 2025 Jun 7;32(6):335. doi: 10.3390/curroncol32060335 (PMC12192035; doi:10.3390/curroncol32060335)
Supplement: Supplementary file 1 [file curroncol-32-00335-s001.zip › Table S2.pdf]

Table S2. Pre- and post-operative LIPI scores divided into upstage and non-upstage

Parameter	Upstage group (n=8)	Non-upstage group (n=72)	p
Pre-operative LIPI score, (%)			0.644
0	7 (87.5)	53 (73.6)	
1	1 (12.5)	15 (20.8)	
2	0 (0)	4 (5.6)	
Post-operative LIPI score, (%)			<.0001
0	0 (0)	67 (93.1)	
1	6 (75)	5 (6.9)	
2	2 (25)	0 (0)	

LIPI, Lung immune prognostic index.
