# Supplementary material for: Increased Pre-Operative Lung Immune Prognostic Index Score Is a Prognostic Factor in Cases of Pathological T3 Renal Cell Carcinoma
Source: Curr Oncol. 2025 Jun 7;32(6):335. doi: 10.3390/curroncol32060335 (PMC12192035; doi:10.3390/curroncol32060335)
Supplement: Supplementary file 1 [file curroncol-32-00335-s001.zip › Table S3.pdf]

Table S3. Some of the previous studies on LIPI and their results.

Study (First Author, Year)	Cancer Type	N (Patients)	LIPI Cut-offs	Prognostic Outcomes	Key Findings
Mezquita et al., 2018 [1]	NSCLC (Non-small cell lung cancer)	297 (ICI-treated)	dNLR >3 and LDH > ULN	OS, PFS	LIPI score predicted ICI response and survival in NSCLC.
Meyers et al., 2019 [6]	Multiple solid tumors	578	dNLR >3 and LDH > ULN	OS, PFS, ORR	The prognostic value of LIPI may be tumor agnostic.
Carril-Ajuria et al., 2024 [8]	Metastatic RCC	1862	dNLR >3 and LDH > ULN	OS, PFS	Pretreatment-LIPI correlated with worse survival outcomes in mRCC.
Obayashi et al., 2022 [9]	Urothelial bladder cancer	105	dNLR >3 and LDH > ULN	OS, DFS, CSS	The preoperative LIPI can predict the prognosis of patients with bladder cancer undergoing radical cystectomy.

CSS, cancer-specific survival; dNLR, Derived neutrophil-to-leukocyte ratio; ICIs, Immune checkpoint inhibitors; LDH, Lactate dehydrogenase; LIPI, Lung Immune Prognostic Index; OS, Overall survival; ORR, overall response rate; PFS, progression-free survival; RCC, Renal cell carcinoma; ULN, upper limit of normal
